# Supplementary material for: Precise in vivo functional analysis of DNA variants with base editing using ACEofBASEs target prediction
Source: eLife. 2022 Apr 4;11:e72124. doi: 10.7554/eLife.72124 (PMC9033269; doi:10.7554/eLife.72124)
Supplement: Supplementary file 1. — Shows nucleotide position (of CDS) and corresponding amino acid with changes. Note: only cytosines on the protospacer with clear editing are shown. *sgRNA on complementary strand [file elife-72124-supp1.docx]

| **Genomic locus targeted** | **Edited cytosine (Amino acid, position, change)** | **Protospacer position (with dinucleotide context)** | **Editing efficiency (Mean ± SD)** | | |
| --- | --- | --- | --- | --- | --- |
|  |  |  | **BE4-Gam** | **ancBE4max** | **evoBE4max** |
| ***O. latipes oca2-Q333*** | C995 > T (T332I) | aC5 | 29.3 ± 6.7 | 86.4 ± 11.5 | 20.7 ± 0.6 |
|  | C996 > T (T332) | cC6 | 38.0 ± 7.9 | 98.2 ± 2.7 | 100 ± 0 |
|  | C997 > T (Q333*) | cC7 | 29.3 ± 7.4 | 93.8 ± 7.9 | 93.3 ± 9.8 |
| ***O. latipes tnnt2a-Q114*** | C337 > T (R113C) | gC5 | 0 ± 0 | 24 ± 14.3 | 26.8 ± 13.2 |
|  | C339 > T (R113) | gC7 | 0 ± 0 | 14.5 ± 15.1 | 65.8 ± 20.6 |
|  | C340 > T (Q114*) | cC8 | 0 ± 0 | 27.8 ± 16.8 | 85.9 ± 23.5 |
| ***O. latipes kcnh6a-Q11*** | C26 > T (A9V) | gC3 | 0.6 ± 1.3 | 3 ± 2 | 29 ± 20.3 |
|  | C28 > T (L10F) | gC5 | 17.8 ± 2.7 | 45.8 ± 15.8 | 95.3 ± 6.7 |
|  | C30 > T (L10) | tC7 | 90.4 ± 13.8 | 92.5 ± 5.8 | 93 ± 7.6 |
|  | C31 > T (Q11*) | cC8 | 61.0 ± 10.4 | 92.8 ± 6.4 | 86.9 ± 15.6 |
| ***O. latipes kcnh6a- R509*** | C1523 > T (A508V) | gC6 | - | - | 92.7 ± 12.8 |
|  | C1524 > T (A508) | cC7 | - | - | 93.3 ± 11.3 |
|  | C1525 > T (R509*) | cC8 | - | - | 39.7 ± 23.9 |
| ***O. latipes kcnh6a- L511*** | C1531 > T (L511F) | gC5 | - | - | 47.5 ± 12.8 |
|  | C1533 > T (L511) | tC7 | - | - | 75.8 ± 9.3 |
| ***O. latipes kcnh6a- R522*** | C1563 > T (D521) | aC6 | - | - | 0 ± 0 |
|  | C1564 > T (R522C) | cC7 | - | - | 54.2 ± 17.0 |
| ***O. latipes tnnt2c-R112*** | C332 > T (A111V) | gC4 | 26 ± 7.1 | - | 60.4 ± 10.4 |
|  | C334 > T (R112*) | aC6 | 2 ± 2.8 | - | 67.6 ± 10.1 |
| ***O. latipes s1pr2-R150**** | C449 > T (R150H) | gC8 | - | - | 21.5 ± 6.9 |
|  | C447 > T (E149) | gC10 | - | - | 30.2 ± 10.5 |
|  | C445 > T (E149K) | tC12 | - | - | 38 ± 20.5 |
| ***O. latipes s1pr2-R167*** | C498 > T (C166) | gC3 | - | - | 24.7 ± 16.5 |
|  | C499 > T (R167C) | cC4 | - | - | 37.7 ± 6.7 |
|  | C501 > T (R167) | gC6 | - | - | 88 ± 12.2 |
| ***O. latipes tnnt2a-W201**** | C603 > T (W201X) | tC4 | - | - | 72.0 ± 34.1 |
|  | C602 > T (W201X) | cC5 | - | - | 73.8 ± 14.9 |
|  | C600 > T (E200) | aC7 | - | - | 4.0 ± 5.5 |
|  | C598 > T (E200K) | tC9 | - | - | 51.4 ± 21.1 |
|  | C597 > T (W199X) | cC10 | - | - | 49.0 ± 8.3 |
|  | C596 > T (W199X) | cC11 | - | - | 22.8 ± 12.5 |
