# Supplementary material for: Precise in vivo functional analysis of DNA variants with base editing using ACEofBASEs target prediction
Source: eLife. 2022 Apr 4;11:e72124. doi: 10.7554/eLife.72124 (PMC9033269; doi:10.7554/eLife.72124)
Supplement: Supplementary file 2. — Shows nucleotide position (of CDS) and corresponding amino acid with changes. Note: only adenines on the protospacer with clear editing are shown. *sgRNA on complementary strand #averaged over single oca2-Q333 and pooled injections [file elife-72124-supp2.docx]

| **Genomic locus targeted** | **Edited adenine (Amino acid, position, change)** | **Protospacer position (with dinucleotide context)** | **Editing efficiency (Mean ± SD)** |
| --- | --- | --- | --- |
| ***O. latipes oca2-Q256*** | A767 > G (Q256R) | cA6 | 100 ± 0 |
|  | A768 > G (Q256) | aA7 | 80 ± 1.7 |
| ***O. latipes oca2-T306*** | A918 > G (T306) | cA4 | 52.3 ± 2.3 |
|  | A919 > G (I307) | aA5 | 4.7 ± 1.2 |
| ***O. latipes oca2-Q333^#^*** | A994 > G (T332A) | aA4 | 96.5 ± 7.6 |
|  | A998 > G (Q333R) | cA8 | 89.7 ± 4.4 |
| ***D. rerio oca2-L293**** | A878 > G (L293P) | cA5 | 52.4 ± 32.9 |
| ***GFP-C71**** | A211 > G (C71R) | cA4 | 97.0 ± 4.4 |
|  | A206 > G (V69A) | cA9 | 61.2 ± 9.4 |
| ***O. latipes kcnh6a-T507*** | A1517 > G (K506) | aA4 | 0.1 ± 0.4 |
|  | A1519 > G (T507A) | gA6 | 81.6 ± 7.2 |
|  | A1521 > G (T507) | cA8 | 44.1 ± 6.2 |
| ***O. latipes kcnh6a-R512*** | A1534 > G (R512G) | cA8 | 92.1 ± 9.2 |
| ***O. latipes kcnh6a-D521*** | A1562 > G (D521G) | gA5 | 61.0 ± 12.5 |
|  | A1568 > G (Y523C) | tA11 | 19.6 ± 2.8 |
| ***O. latipes tnnt2a-e2-SA*** | A41(-2) > G (SA) | cA4 | 6.5 ± 5.4 |
|  | A41 > G (D14G) | gA6 | 72.5 ± 8.3 |
| ***O. latipes tnnt2a-D189G;R190G*** | A566 > G (D189G) | gA6 | 11.5 ± 13.0 |
|  | A568 > G (R190G) | tA8 | 28.5 ± 24.4 |
