# Supplementary material for: Precise in vivo functional analysis of DNA variants with base editing using ACEofBASEs target prediction
Source: eLife. 2022 Apr 4;11:e72124. doi: 10.7554/eLife.72124 (PMC9033269; doi:10.7554/eLife.72124)
Supplement: Supplementary file 3. — Shows nucleotide position (of CDS) and corresponding amino acid with changes. Note: only cytosines on the protospacer with clear editing are shown. *sgRNA on complementary strand [file elife-72124-supp3.docx]

| **Genomic locus targeted** | **Edited cytosine (Amino acid, position, change)** | **Protospacer position (with dinucleotide context)** | **evoBE4max editing efficiency (Mean ± SD)** |
| --- | --- | --- | --- |
| ***O. latipes dapk3-P204 (n=7)*** | C610 > T (P204L) | gC5 | 64.7 ± 16.3 |
|  | C611 > T (P204L) | cC6 | 86.9 ± 8.7 |
|  | C613 > T (L205F) | gC8 | 61.7 ± 14.1 |
| ***O. latipes ptpn11-G504* (n=11)*** | C1511 > T (G504R/K) | cC5 | 87.4 ± 7.7 |
|  | C1510 > T (G504E/K) | cC6 | 88.0 ± 7.3 |
| ***O. latipes ube2b-R8* (n=5)*** | C23 > T (R8Q) | tC5 | 54.2 ± 24.8 |
|  | C20 > T (R7K) | tC8 | 67.4 ± 16.8 |
| ***O. latipes usp44-E68* (n=11)*** | C202 > T (E68K) | tC7 | 99.7 ± 0.9 |
|  | C201 > T (M67I) | cC8 | 97.0 ± 5.0 |
