# Supplementary material for: Precise in vivo functional analysis of DNA variants with base editing using ACEofBASEs target prediction
Source: eLife. 2022 Apr 4;11:e72124. doi: 10.7554/eLife.72124 (PMC9033269; doi:10.7554/eLife.72124)
Supplement: Supplementary file 4. — Comparison of target nucleotide editing efficiency and overview of indel frequency from Illumina data. [file elife-72124-supp4.docx]

| **Locus tested** | **Base editor used** | **Target C or A to T or G editing efficiency (%) (Mean ± SD)** | | **Indel frequency (%)** |
| --- | --- | --- | --- | --- |
|  |  | **Sanger sequencing + EditR** | **Amplicon sequencing (Illumina)** | **Amplicon sequencing (Illumina)** |
| oca2-Q333 | BE4-Gam | 29.3 ± 7.4 | 25.9 | 2.1 |
|  | ancBE4max | 93.8 ± 7.9 | 65.3 | 19.9 |
|  | evoBE4max | 93.3 ± 9.8 | 79.1 | 14.4 |
|  | ABE8e | 89.7 ± 4.4 | 79.2 ± 4.6 | 8.0 ± 2.3 |
| GFP-C71 | ABE8e | 97.0 ± 4.4 | 85.9 ± 15.3 | 15.8 ± 17.5 |
| tnnt2a-Q114 | evoBE4max | 85.9 ± 23.5 | 89.3 ± 6.6 | 7.7 ± 7.3 |
| kcnh6a-R512 | ABE8e | 92.1 ± 9.2 | 86.1 ± 15.8 | 14.2 ± 15.4 |
| ube2b-R8 | evoBE4max | 54.2 ± 24.8 | 46.6 ± 11.9 | 4.9 ± 4.3 |
|  | | | | |
| evoBE4max summary (three loci) | | 77.8 ± 20.8 | 71.7 ± 22.3 | 9.0 ± 4.9 |
| ABE8e summary (three loci) | | 92.9 ± 3.7 | 83.7 ± 3.9 | 12.7 ± 4.1 |
